# A functional variant alters binding of activating protein 1 regulating expression of *FGF7* gene associated with chronic obstructive pulmonary disease

**DOI:** 10.1186/s12881-019-0761-7

**Published:** 2019-02-18

**Authors:** Xiaomei Zhang, Yongxin Guo, Jing Yang, Jianlou Niu, Lina Du, Haiyan Li, Xiaokun Li

**Affiliations:** 1 College of Life Science, Engineering Research Center of the Chinese Ministry of Education for Bioreactor and Pharmaceutical Development, Jilin Agricultural University, NO. 2888, XinCheng Avenue, Changchun, 130118 China; 2 School of Pharmacy, Wenzhou Medical University, Chashan Avenue, Wenzhou, 325035 Zhejiang China

**Keywords:** FGF7, COPD, AP-1

## Abstract

**Background:**

Genome-wide association studies (GWASs) of a large cohort of subjects with chronic obstructive pulmonary disease (COPD) have successfully identified multiple risk genes, including fibroblast growth factor 7 (*FGF7*). However, the underlying molecular mechanism influencing function of FGF7 and risk of COPD remains further study.

**Methods:**

In this study, we replicated the genetic association of variants near the *FGF7* gene in 258 Chinese Han patients with COPD and 311 healthy controls. Additionally, we functionally evaluated a candidate causal variant upstream of the FGF7 gene.

**Results:**

The most significant association was observed at rs12905203 (*P* = 5.9 × 10^− 3^, odd ratio, OR = 1.516) that explains associations of previously reported variants at the *FGF7* locus. Electrophoretic mobility shift assay (EMSA) and chromatin immunoprecipitation-quantitative polymerase chain reaction (ChIP-qPCR) assays showed that the risk allele of the variant was bound to activator protein 1 transcription factors (c-Fos and c-Jun) with a significantly reduced affinity and associated with decreased mRNA expression of *FGF7* in fibroblast cells at both resting and PMA/Ionomycin-stimulated conditions. Overexpression of c-Fos and c-Jun proteins or stimulation with PMA/Ionomycin significantly increases mRNA expression of *FGF7* in fibroblast cells. Bioinformatic analysis showed that the variant overlaps with multiple genetic regulatory marks, suggesting the regulatory DNA element might function as an enhancer for the *FGF7* gene. Luciferase enhancer activity assays demonstrated that the DNA sequences carrying the variant produce enhancer activity while the risk allele of the variant reduces its activity.

**Conclusions:**

In this study, we demonstrated a consistent association of the *FGF7* gene with COPD and mechanistically characterized a candidate functional variant upstream of the *FGF7* gene. These data highlighted the important role of the risk variant and the *FGF7* gene in influencing risk for COPD.

**Electronic supplementary material:**

The online version of this article (10.1186/s12881-019-0761-7) contains supplementary material, which is available to authorized users.

## Background

Chronic obstructive pulmonary disease (COPD) is a complex genetic disorder that is characterized by a reduction in lung function with airflow obstruction [1]. Although cigarette smoking is a common risk factor for COPD, cigarette smoking can differentially affect lung function, and not all cigarette smokers develop COPD [2–4]. The response to cigarette smoking as well as other environmental factors is significantly influenced by a complex array of genetic factors [1, 5]. Four large-scale genome-wide association studies have been performed in multiple populations and successfully identified numerous genetic variants consistently associated with COPD (Additional file 1: Table S1) [6–9]. In addition to the GWAS, candidate gene approaches also significantly contributed to the identification of risk factors of COPD in the individual cohort of patients [10–12].

The *FGF7* gene encodes keratinocyte growth factor (KGF), a member of the FGF family that are involved in various biological processes, including embryonic development, morphogenesis, cell growth, tumor growth, and tissue repair [13, 14]. Recent studies have demonstrated a significant association of genetic variants at the *FGF7* gene in COPD patients of Spanish, Native American, Norwegian (2940 cases and 1380 controls in total, rs12591300 and rs4480740) [10], and Chinese Han (279 cases and 367 controls in total, rs10519225) ancestry [12]. Due to the relatively small sample size in the Chinese Han study, further evaluation of the genetic association of the *FGF7* gene in an independent cohort of Chinese COPD is needed.

KGF, encoded by the *FGF7* gene, is mainly related to the repair of the lung, and that is mostly due to their capacity to stimulate alveolar and bronchial epithelial cell proliferation [15, 16]. Although the potential role of *FGF7* in influencing the risk of COPD is poorly understood, functional studies have been performed to investigate gene expression abnormalities of the *FGF7* in patients with COPD [17]. A study showed that the KGF levels were not notably different between patients with COPD and healthy controls in bronchoalveolar lavage (BAL) fluid or in serum, which may be due to the limitation of the KGF detection method used in the samples [17]. Also, studies on the role of human recombinant KGF in modulating lung function have also been conducted in cell-based assays and mouse models. The expression of KGF increases after lung injury in humans and minimizes lung injury in experimental animals [18, 19]. These data further suggested an essential role of fibroblast growth factor signaling as well as the KGF protein in the development and the treatment of COPD [14, 15, 18, 20, 21].

Human genetic variations and epigenetic mechanisms play a critical role in regulating the expression of the *FGF7* gene. Further assessment of genetic association and mechanistic characterization of the COPD-associated functional variants of the *FGF7* gene are critical steps to understand the disease mechanisms. In the current study, therefore, we employed a combination of approaches, including bioinformatic analysis of candidate functional variants, functional evaluation of transcription factor binding of variant by electrophoretic mobility shift assay (EMSA), gene expression assays of *FGF7* using real-time quantitative polymerase chain reaction (RT-qPCR), chromatin conformation capture followed by RT-qPCR (3C-qPCR), and luciferase enhancer activity assays to characterize the COPD-associated candidate causal variant. The current study provides significant insight into the functional variants of the *FGF7* gene in influencing risk for COPD.

## Methods

### Subjects

In this study, a total of 258 patients with COPD and 311 matched non-COPD population controls were enrolled. The control subjects were healthy donors with Chinese Han descent. The subjects in both groups were self-reported Chinese Han individuals and were recruited from Wenzhou in the Zhejiang Province of China. Each subject was interviewed by interviewers who collected the patients’ demographic data and information related to risk factors, such as smoking cigarettes. The clinical analyses were performed at Wenzhou Medical University; diagnosis of patients was performed according to the Global Initiative for Chronic Obstructive Lung Disease (GOLD) criteria. All patients were subjected for clinical testing and evaluation of genetic variations. Patients were excluded from this study if they had other respiratory diseases (bronchial asthma, bronchiectasis, cancer, or pulmonary tuberculosis). Control subjects were excluded if they had a history of lung disease, atopy, an acute pulmonary infection in the 4 weeks before the enrolment for this study. This study was approved by Institutional Review Board of Jilin Agricultural University and Ethics Committee of the Second Affiliated Hospital, Wenzhou Medical University. The investigator explained the purpose and risks of the study and provided the subject with a copy of the information sheet.

### SNP selection

Based on the LD and haplotype block analysis using HaploReg 4, we included 68 SNPs in LD with the three tag SNPs with an *r*^2^ 0.8 for an initial screening. Further bioinformatic analyses of the list of SNPs were performed to exclude predicted SNP motif changes less than 5. By applying the filter, 17 SNPs remained for further analysis. Because a SNP rs12905203 is expected to change 19 binding motifs for multiple transcription factors, we included this candidate functional variant along with the reported Asian COPD-associated variant for this genetic association study.

### SNP genotyping

Isolation of genomic DNA from PBMCs was performed using a DNA extraction and purification kit (TAKARA, Dalian, China), according to the manufacturer’s instructions. Sequences of the PCR primers are listed in Additional file 3: Table S3. The genotypes at selected SNPs for each genomic DNA sample with PCR amplified target regions were determined using a TaqMan SNP genotyping assay kit (Thermo Fisher Scientific Inc., Waltham, MA, USA). The assay followed the protocol in the manual, and PCR reactions were conducted on an OpenArray™ real-time PCR instrument (Applied Biosystems™, Foster City, CA, USA).

### Statistical analysis

The demographic and clinical data between the COPD patients and the controls were analyzed using the chi-square test and Student’s t-test. Hardy-Weinberg equilibrium was calculated with a goodness of fit chi-squared test to assess the observed and expected genotype frequencies. The differences between the COPD patients and the control subjects in the context of the genotypes were analyzed using one-way analysis of variance and logistic regression for multivariate analysis. Age, sex, and smoking were used as covariates in the multivariate analyses. Statistical analyses were conducted using SPSS version 21.0 and Microsoft Excel.

### Antibodies, plasmid DNAs, and cell lines

Expression constructs expressing human c-Fos (pLX304-FOS-V5) and c-Jun (pCLXSN-c-JUN) were purchased from Addgene (Addgene Headquarters, Cambridge, MA, USA). Luciferase activity assay backbone DNA pGLuc-mini-TK was purchased from New England Biolabs (Ipswich, MA, USA). Anti-KGF, anti-c-Fos, and anti-c-Jun antibodies were purchased from Abcam (Abcam, Inc. Shanghai, China). Sixty-nine skin fibroblast cell lines were established from healthy Chinese donors.

### RNA isolation and quantitative RT-PCR

Total RNA from fibroblast cell lines was isolated using a Total RNA isolation and purification kit (Invitrogen Inc., Carlsbad, CA, USA) according to the manufacturer’s instructions. cDNA from each sample was synthesized using iScript cDNA Synthesis Kits (Bio-Rad Laboratories, Inc., Hercules, CA, USA). Quantitative RT-PCR assays were performed using Power SYBR Green Master Mixture (Applied Biosystems™, Foster City, CA, USA) to determine the mRNA expression of the FGF7 gene in each sample, and glyceraldehyde 3-phosphate dehydrogenase (GAPDH) was used as the internal control. Fold changes were calculated according to the ΔΔCT method.

### Electrophoretic mobility shift assays

Fifty base pair non-risk or risk DNA probes were synthesized and end-labeled with a Biotin-DNA labeling kit (Thermo-Fisher). Nuclear protein extracts were prepared from fibroblast cells transfected with plasmid DNA expressing c-Fos, c-Jun, or control vector. A portion of the cells was stimulated with PMA/Ionomycin (50 ng/ml, 500 ng/ml) for 2 h. Incubation of nuclear proteins with labeled probes was performed for 25 min at 37 °C in the binding buffer (1 μg poly dI-dC, 20 mM HEPES, 10% Glycerol, 100 mM KCl, and 0.2 mM EDTA, pH 7.9). DNA-protein complexes were resolved on non-denaturing acrylamide gels.

### ChIP and qPCR

ChIP assays were performed using the Millipore Magna ChIP A kit (Millipore, Billerica, CA, USA) according to the manufacturer’s instructions. The chromatin-protein complexes were immune-precipitated by antibodies specific for c-Fos, c-Jun, or rabbit IgG (negative control) overnight at 4 °C. DNA was eluted from the immune-precipitated chromatin complexes, reverse-crosslinked, purified by a DNA purification kit (TAKARA, Dalian, China), and subjected to real-time PCR analysis. Sequences of primers are listed in the Additional file 4: Table S4

### Luciferase assay

We cloned 200 bp of DNA sequence surrounding the variant rs12905203 into a minimal TK promoter luciferase plasmid. Each plasmid was transiently co-transfected with a pGL3-promoter transfection efficiency control plasmid for normalization. Luciferase assays were performed in fibroblast cells. Forty eight hours post-transfection, cells were treated with 50 ng/ml PMA/500 ng/ml Ionomycin for additional 48 h. The cells were lysed using a lysis butter provided by the Kit, and the luciferase activity was measured using the Luciferase Assay System (Promega, Madison, MA, USA).

## Results

### Genetic variants at the *FGF7* locus associated with COPD in Chinese Han

**Fig. 1:**
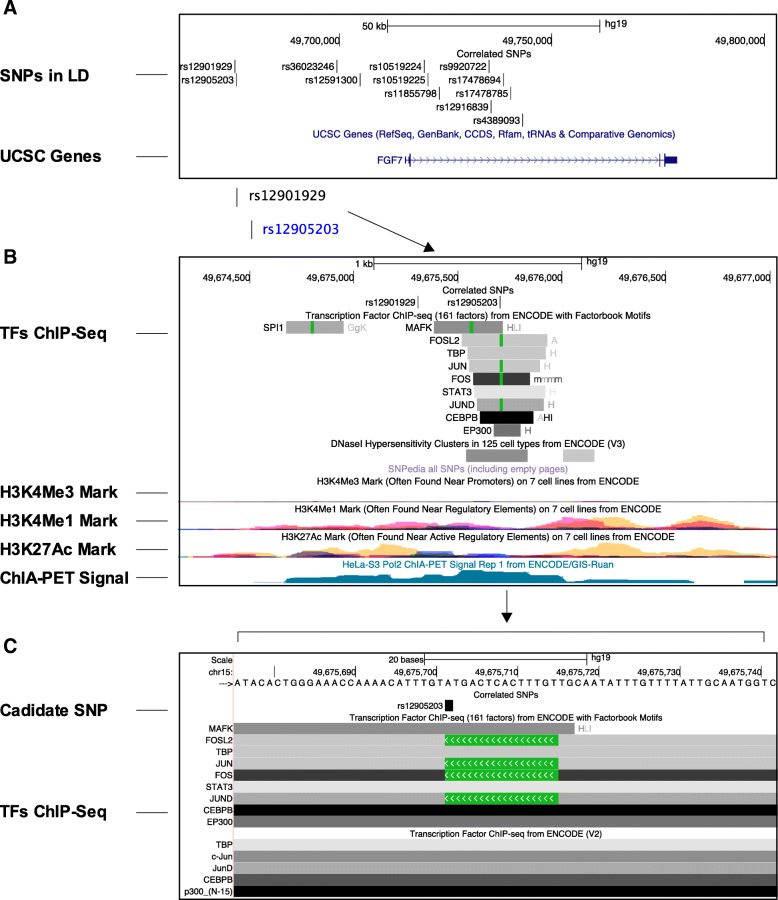
The candidate functional variant resides in a putative enhancer element. **a** the relative locations of the 12 SNPs that correlated with the tag-SNPs rs12591300 (or rs10519225) with an *r*^2^ greater than 0.8. Four SNPs lie upstream of the promoter of the *FGF7* gene, and eight SNPs are in the intron of the *FGF7* gene. **b** The variant rs12905203 resides in a putative enhancer element that binds multiple transcription factors, including AP-1 and histone modification enzymes, and overlaps with H3K4me1 and H3K27ac marks. **c** Zoomed in view of the variant and surrounding DNA sequence revealed that the altered allele of the variant might affect binding of AP-1 transcription factors

Three SNPs have demonstrated significant associations with COPD, two of which have been identified in Spanish, Native American ancestry (rs12591300 and rs4480740). The variant rs10519225 showed an association in COPD in Chinese Han individuals [12]. Using HaploReg [22], we identified 10 single nucleotide polymorphisms (SNPs) that are in strong linkage disequilibrium (LD) with risk variants rs10519225 and rs12591300 (*r*^2^ > 0.8) (Additional file 2: Table S2). Genetic variants commonly regulate gene expression through alteration of DNA sequences that contain binding motifs for transcription factors. Therefore, we prioritized candidate SNPs for replication of genetic association and functional evaluation based on predicted motif changes. As shown in Additional file 2: Table S2, an *FGF7* gene upstream variant rs12905203 was expected to change the binding sequences for 19 transcription factors reasoning that the rs12905203 is likely a candidate functional variant (Additional file 2: Table S2). Therefore, we included the candidate functional variant rs12905203 along with the previous reported Asian COPD associated-variants (rs10519225 and rs4480740) in this genetic association study. We genotyped the three variants in 258 COPD cases and 311 matched controls (Table 1). The most significant association was observed at the variant rs12905203 (*P* = 5.9 × 10^− 3^, OR = 1.516) (Table 2). A conditional analysis was then performed on the rs12905203; there was no residual association observed for the rs10519225 and rs4480740. We further evaluated the functional potential of the rs12905203 using the table browser tool integrated into the UCSC genome browser by searching for positional overlapping with transcription factor binding sequences and histone modification marks [23, 24]. As shown in Fig. 1, the variant lies on a DNA sequence that binds to multiple transcription factors, including MAFK, FOSL2, TBP, JUN, FOS, STAT3, JUND, CEBPB, and EP300. The variant also overlaps with H3K4me1 and H3K27Ac marks suggesting that the regulatory DNA elements might function as an enhancer upstream of the *FGF7* gene.

**Table 1:** Description of study population

Variable		Controls	Cases	*P*
		(*n* = 311)	(*n* = 258)	
Age, years		63 ± 9	62 ± 8	NS
Sex(Male/Female)		277/34	230/28	NS
Smoking history	0–20 pack years	69	54	NS
	20 pack years	242	204	NS
FEV1		1.81 ± 0.96	0.92 ± 0.45	< 0.05
FEV1 percentage of predicted, %		91.5 ± 4.8	44.1 ± 0.51	< 0.05
FEV1/FVC, %		76.2 ± 5.7	44.8 ± 9.4	< 0.05

*FEV1* Forced Expiratory Volume in 1 s, *NS* not significant. Data are presented as mean ± SEM. *FVC* Forced Vital Capacity

**Table 2:** Associations of rs10519225 and rs10519255 with COPD

Genotype	Frequency			
	Controls	COPD cases	OR (95% CI)	*P* value
rs12905203 A/G				
AA	205	138	1.516 (1.127–2.038)	0.005
AG	96	110		
GG	10	10		
MAF	0.1865	0.2519		
rs10519225 G/A				
GG	211	148	1.427 (1.048–1.943)	0.024
GA	92	104		
AA	8	6		
MAF	0.1736	0.2248		
rs4480740 G/A				
GG	222	168	1.237 (0.8958–1.708)	0.196
GA	82	86		
AA	7	4		
MAF	0.1822	0.1543		

Statistically significance was calculated using logistic regression with an additive model for both variants

### The COPD-associated risk allele of the candidate functional variant reduces binding to the c-Fos and c-Jun transcription factors

**Fig. 2:**
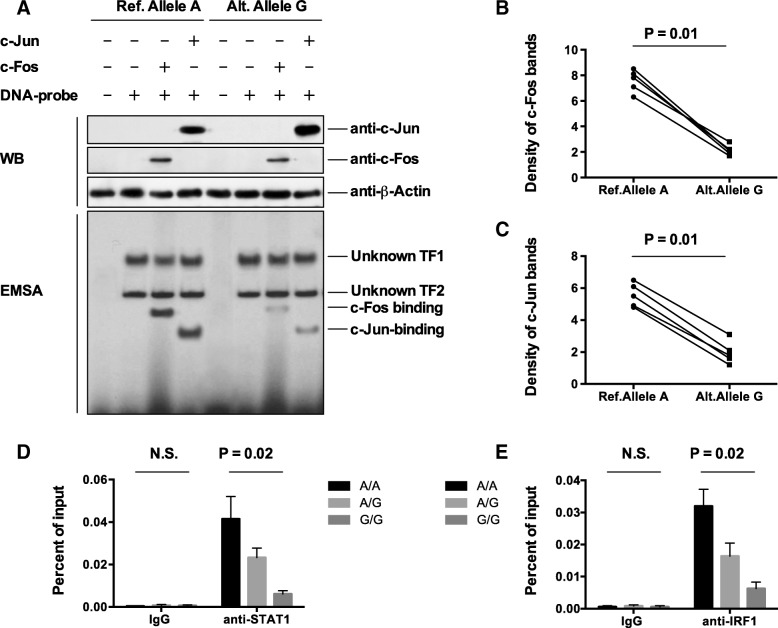
The risk allele of the variant binds AP-1 subunits with reduced affinity. **a** A representative figure from three independent western blotting experiments demonstrated the expression of the AP-1 subunits, c-Fos, and c-Jun in fibroblast cells. EMSA revealed that multiple nuclear protein complexes bind to the variant region in which the risk allele of the variant results in reduced binding of the AP-1 subunits. **b** and **c** We performed densitometric quantification of AP-1 subunits binding in independent experiments for c-Fos (**b**) and c-Jun cells (**c**). Statistical comparisons were made using paired Student’s t-test. **d** and **e** ChIP-qPCR was performed using fibroblast cell lines carrying different genotypes of the variants (AA, AG, and GG). ChIP was performed with antibodies specific against c-Fos and c-Jun subunits, respectively, followed by qPCR with primers neighboring the variant rs12905203 region. Statistical comparisons were made using one-way ANOVA

**Fig. 3:**
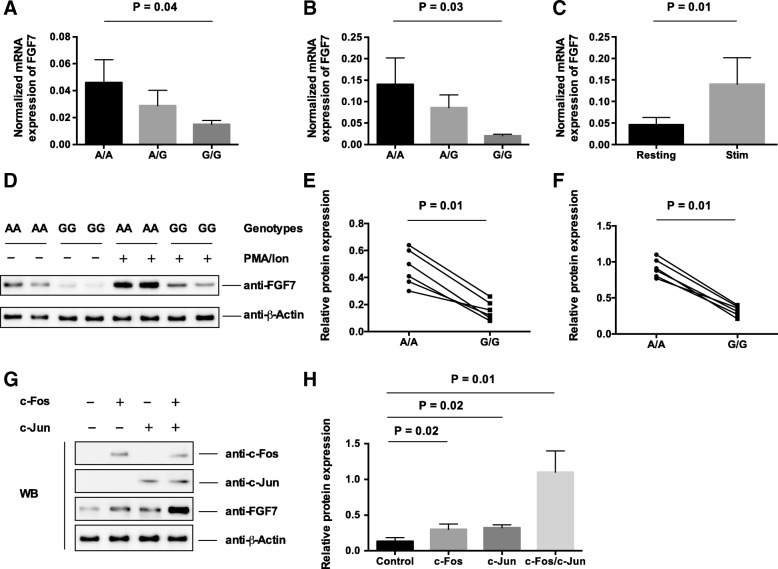
Overexpression of AP-1 transcription factors or stimulation with PMA/Ionomycin increases expression of the *FGF7* gene in fibroblast cells. **a** and **b** Messenger RNA expression of the *FGF7* gene in fibroblast cell lines carrying various genotypes at the variant rs12905203 at resting- and stimulated-conditions. **c** mRNA expression of the *FGF7* gene in fibroblast cell lines at resting and stimulated conditions. **d** Protein expression of the KGF in fibroblast cell lines carrying homozygous risk and non-risk variants was determined using western blotting with an anti-KGF antibody. **e** and **f** The protein expression of KGF in fibroblast cell lines were normalized to the beta-actin controls for resting- (**e**) and stimulated (**f**) conditions. Three independent experiments were performed, and the differences in protein expression of the KGF were calculated by paired Student’s t-test. **g** Fibroblast cells were transfected with c-Fos, c-Jun, or c-Fos/c-Jun expression vectors. The expression of the KGF protein was determined by western blotting with an antibody against KGF. **h** We performed densitometric quantification of c-Fos, c-Jun, and KGF in three independent experiments. The statistical differences between groups were calculated by using Student’s t-test

Because the variant lies on the consensus motifs for FOS and JUN (as shown in Additional file 2: Table S2 and Fig. 1), we assessed the binding of the variant to the c-Fos and c-Jun transcription factors using EMSA and chromatin immunoprecipitation-quantitative polymerase chain reaction (ChIP-qPCR) assays. As shown in Fig. 2a, the c-Fos and c-Jun proteins were successfully overexpressed in fibroblast cells. EMSA assays demonstrated that the DNA probe carrying the non-risk allele (A) of the variant binds to both c-Fos and c-Jun, while the DNA probe carrying the risk allele (G) of the variant showed a significant reduction of binding in vitro (Figs. 2b and 3c). To further evaluate the observation in EMSA assays, we performed ChIP-qPCR assays with antibodies against c-Fos and c-Jun in fibroblast cells, respectively. As shown in Fig. 2d and e, the transcription factors, c-Fos and c-Jun, were significantly enriched at the variant locus. These data suggested that the AP-1 transcription factors bind to the variant, and the allelic differences in transcription factor binding might influence the transcriptional activity of the putative variant-containing enhancer upstream of the *FGF7* gene.

### The COPD-associated risk allele of the variant correlated with a reduction of mRNA expression in fibroblast cell lines

To further assess the functional potential of the variant in regulating mRNA expression of the *FGF7* gene, we determined the mRNA expression of the *FGF7* by RT-qPCR in 69 fibroblast cell lines derived from healthy donors. Individuals carrying the COPD-associated risk allele displayed a significant reduction of the *FGF7* gene in normal culture conditions (Fig. 3a) as well as after stimulation with PMA/Ionomycin (Fig. 3b). Additionally, we showed that the expression of the *FGF7* is significantly increased after stimulation with PMA/Ionomycin (Fig. 3c). To further evaluate the role of the variant in regulating protein expression of the KGF, we measured protein expression by western blotting with an antibody against KGF. As shown in Fig. 3d, e, and f, the protein expression of KGF is increased after stimulation with PMA/Ionomycin. Consistent with the mRNA expression, we observed that individuals carrying the risk allele show a significant decrease in protein expression of KGF.

### Over-expression of AP1 transcription factor increases the FGF7 expression in fibroblast cells

To evaluate the role of AP-1 transcription factors in regulating the protein expression of KGF, we performed western blotting using fibroblast cells transfected with plasmid DNAs encoding the c-Fos and c-Jun transcription factors, respectively. As shown in Fig. 3g and h, overexpression of c-Fos or c-Jun, was able to increase the protein expression of KGF. Additionally, co-transfection of the cells with c-Fos expressing vector and c-Jun expressing vector increases protein expression of KGF (*P* = 0.0007). These data highlighted an essential role of the AP-1 transcription factors in regulating *FGF7* expression in the fibroblast cells.

### The regulatory DNA element carrying the COPD-associated variant functions as an enhancer

**Fig. 4:**
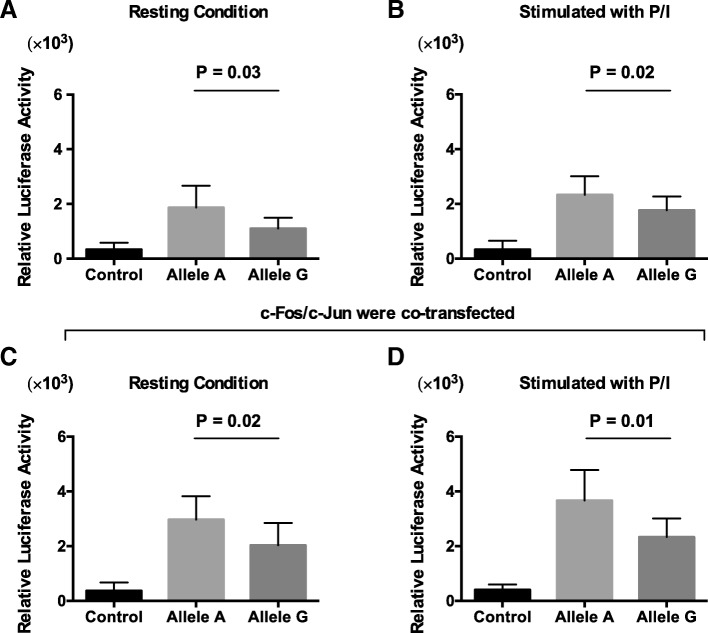
The regulatory element containing the rs12905203 variant demonstrates enhancer activity. **a** and **b** Sequences carrying risk (G) and non-risk (**a**) variants were cloned upstream of a minimal thymidine kinase promoter luciferase construct to measure luciferase activation following transient transfection and stimulation with PMA/Ionomycin. **c** and **d** The luciferase activity assay vectors were co-transfected with constructs expressing AP-1 transcription factors. Seventy-two hours post-transfection, luciferase activity for risk and non-risk variants was measured and compared to the control. The statistical differences between risk and non-risk alleles of the variant were calculated using Student’s t-test

Because the variant binds to AP-1 transcription factors and overlaps with various enhancer marks, we hypothesized that the regulatory DNA element carrying the variant might be an upstream enhancer of the *FGF7* gene. To test this hypothesis, we cloned a 200-bp DNA sequence centered at the variant into a minimal TK promoter construct. Plasmids were transfected into fibroblast cells followed by stimulation with PMA/Ionomycin. Compared to the minimal TK promoter alone, we observed a significant increase in luciferase activity following stimulation with PMA/Ionomycin for both non-risk (A) and risk (G) plasmids, suggesting that this regulatory element functions as an enhancer. However, the risk (G) construct produced significantly lower levels of luciferase activity compared with the non-risk (A) construct (Fig. 4). These results demonstrated that the regulatory element containing the COPD-associated variant functions as an enhancer, and the presence of the risk (G) allele, which binds AP-1 transcription factors with reduced affinity, impairs enhancer functions.

## Discussion

In this report, we used a combination of approaches, including genetic association testing and bioinformatic analysis to fine-map the COPD-associated variants in *FGF7* locus. Three SNPs have shown significant associations of the *FGF7* in COPD (rs4480740, rs12591300, and rs10519225) [10, 12]. We evaluated SNPs at the *FGF7* locus that are in LD with the reported SNPs and selected candidate functional SNPs for genetic association testing. We demonstrated that multiple variants were significantly associated with Chinese COPD while the most significant associated SNP is rs12905203, located upstream of the promoter of the *FGF7* gene. To our knowledge, this is the second report on the genetic association between the *FGF7* gene and COPD in Chinese Han. In this study, we showed that previously reported COPD-associated risk variants are in strong LD with the functional variant rs12905203. These data suggested a single effect at the FGF7 locus is associated with COPD in Chinese Han. A drawback of our study is of the failure to detect genetic associations of SNPs those were not in LD with the reported tag-SNPs, while there might be additional independent association signals. Additionally, significant associations of the *FGF7* gene with COPD in this study might prove to be falsely positive due to the relatively small sample size, but even with a larger sample, functional characterization of the variant would be required.

The *FGF7* gene, which encodes KGF, was initially identified in cultured human embryonic lung fibroblasts [10, 16]. The gene plays a vital role in protecting airway epithelium from oxidant injury that is potentially related to the pathogenesis of COPD [10, 16]. Genetic alterations of the *FGF7* gene could affect the expression of the gene or function of the encoded protein, KGF. Previous studies demonstrated that FGF7 gene plays an important role in protecting against oxidative stress response. Increases in *FGF7* expression associated with disease severity may indicate a higher burden of injury [18, 19, 25]. We hypothesize that the risk G allele of the functional variant resulted in a decreased expression of *FGF7* and that might influence antioxidant mechanisms protecting against deleterious effects of smoking on the lung. Furthermore, it was unclear whether the *FGF7* gene plays a role in disease susceptibility through its role in epithelial development by influencing epithelial responses to cigarette smoke [2, 3, 13, 25]. Because it is unclear whether increased FGF7 expression is a marker of exposure to oxidant injury or a cause of epithelial damage, we further characterized the role of candidate functional SNP rs12905203 on FGF7 expression.

The most significant association variant lies immediately upstream of *FGF7* in a putative enhancer element. The variant results in a substitution of the consensus binding sequences for multiple transcription factors, including AP-1 [22]. We functionally assessed binding of the variant to AP-1 transcription factors and demonstrated that the COPD-associated risk G allele significantly reduces the binding that is accompanied by a reduction of mRNA expression of the FGF7 gene in fibroblast cells. Stimulation of the fibroblast cells with PMA/Ionomycin or overexpression of AP-1 transcription factors significantly increases the expression of *FGF7* gene, suggesting a role of the AP-1 transcription factors in regulating the transcription of FGF7.

In conclusion, we investigated the association between the *FGF7* variants and the risk of COPD in the Chinese Han population. The variant rs12905203 resides in an enhancer element that binds c-Fos, and c-Jun transcription factors promote the expression of *FGF7*. Impaired binding of c-Fos and c-Jun proteins to the risk allele of the rs12905203 inhibits the activity of the enhancer, resulting in reduced KGF expression. These results provide genetic and functional evidence supporting a causal role for the variant rs12905203 in the genetic predisposition to COPD.

## Conclusion

In this study, we assessed genetic association of FGF7 variant rs12905203 in a new established-COPD cohort in Chinese Han. We identified a functional variant that modulates the gene expression of FGF7 through the regulation of AP-1 binding.

## Additional files

Additional file 1: **Table S1.** GWAS identified COPD-associated risk loci Additional file 5. (PDF 131 kb)

Additional file 2: **Table S2.** Twelve SNPs in strong LD with the tag SNP rs10519225. (PDF 68 kb)

Additional file 3: **Table S3.** The candidate functional variant lies on binding sites for multiple transcription factors. (PDF 71 kb)

Additional file 4: **Table S4.** List of primer sequences for quantitative PCR assays. (PDF 52 kb)

Additional file 5: List of references in Additional file 1: Table S1. (PDF 70 kb)

## Abbreviations

3C-qPCR: Chromatin conformation capture followed by RT-qPCR
ChIP-qPCR: Chromatin immunoprecipitation-quantitative polymerase chain reaction
COPD: Chronic obstructive pulmonary disease
EMSA: Electrophoretic mobility shift assay
FGF7: Fibroblast growth factor 7
GOLD: Global Initiative for Chronic Obstructive Lung Disease
GWAS: Genome-wide association studies
KGF: Keratinocyte growth factor
