# Supplementary material for: A functional variant alters binding of activating protein 1 regulating expression of FGF7 gene associated with chronic obstructive pulmonary disease
Source: BMC Med Genet. 2019 Feb 18;20:33. doi: 10.1186/s12881-019-0761-7 (PMC6380023; doi:10.1186/s12881-019-0761-7)
Supplement: Supplementary file 1 — Table S1. GWAS identified COPD-associated risk loci Additional file 5. (PDF 131 kb) [file 12881_2019_761_MOESM1_ESM.pdf]

Supplementary Table 1 GWAS identified COPD-associated risk loci.

Susceptibility locus	Population information	Sample size	Reference
CHRNA3<br>CHRNA5<br>IREB2	GWAS of COPD in Norway with replication of top associated variants in additional cohorts (ICGN, NETT-NAS, and BEOCOPD)	1,633 participants	Pillai SG, et al. (2009)[1]
FAM13A	GWAS of COPD in Norway, NETT-NAS, and ECLIPSE. Replication was performed in additional cohort (COPDGene, BEOCOPD and ICGN)	2940 cases and 1380 smoking controls	Cho MH, et al. (2010)[2]
BICD1	GWAS of percent emphysema detected by computed tomography (CT) in the Norway, ECLIPSE, and NETT studies.	2,380 patients with COPD	Kong X, et al. (2011)[3]
FTO	GWAS of body mass index (BMI) in ~ in three cohorts: ECLIPSE, Norway, and NETT, with replication attempted in 502 subjects in COPDGene. A GWAS of fat-free mass index (FFMI) in COPD subjects was conducted in ECLIPSE and Norway	3,000 COPD subjects	Wan E, et al. (2011)[4]
FGF7	Norway, NETT, NAS, ECLIPSE	2,940 cases and 1,380 controls	Brehm JM, et al. (2011)[5]
RIN3<br>MMP12<br>TGFB2	GWAS of COPD in cohorts: ECLIPSE, NETT/NAS, and GenKOLS (Norway)	6,633 moderate-to-severe cases and 5,704 controls	Cho MH, et al. (2014)[6]
LINGO2<br>RETNLB<br>SGCD<br>PDSS2	The multicenter LHS cohort	2,657 cases and 2,814 controls	Hansel NN, et al. (2015)[7]

ICGN = International COPD Genetics Network, NETT = National Emphysema Treatment Trial, NAS = Normative Aging Study, BEOCOPD = Boston Early-Onset COPD, Genetic Epidemiology of COPD: COPDGene Study, HRCT = high resolution computed tomography of the thorax, LHS = Lung Health Study

References are listed in the additional file 5
