# Supplementary material for: A functional variant alters binding of activating protein 1 regulating expression of FGF7 gene associated with chronic obstructive pulmonary disease
Source: BMC Med Genet. 2019 Feb 18;20:33. doi: 10.1186/s12881-019-0761-7 (PMC6380023; doi:10.1186/s12881-019-0761-7)
Supplement: Supplementary file 2 — Table S2. Twelve SNPs in strong LD with the tag SNP rs10519225. (PDF 68 kb) [file 12881_2019_761_MOESM2_ESM.pdf]

Supplementary Table 2 Twelve SNPs in strong LD with the tag SNP rs10519225

Chr	Pos (hg38)	LD (r^2)	LD (D')	Variant	Ref	Alt	AFR freq	AMR freq	ASN freq	EUR freq	Enhancer histone marks	DNAse	Proteins bound	Motifs changed
15	49383111	0.97	0.98	rs12901929	T	C	0.05	0.25	0.16	0.38	6 tissues	CRVX		Irf,NF-AT,Pbx-1
**15**	**49383505**	**0.9**	**0.98**	**rs12905203**	**A**	**G**	**0.11**	**0.27**	**0.16**	**0.4**	**6 tissues**	**8 tissues**	**5 bound proteins**	**19 altered motifs**
15	49407114	0.99	1	rs36023246	A	G	0.05	0.25	0.16	0.38		LIV		CAC-binding-protein
15	49412544	0.88	1	rs12591300	G	A	0.05	0.25	0.16	0.35		MUS		Ets,GR
15	49427756	0.99	1	rs10519224	A	C	0.05	0.25	0.16	0.38		HRT,MUS		Foxc1
15	49428581	1	1	rs10519225	G	A	0.05	0.25	0.16	0.38		8 tissues		Arid5b,Pou2f2
15	49431258	0.99	1	rs11855798	G	C	0.05	0.25	0.16	0.38	4 tissues			7 altered motifs
15	49442915	0.93	-0.97	rs9920722	C	T	0.56	0.71	0.79	0.62	5 tissues			5 altered motifs
15	49443468	0.86	0.99	rs12916839	C	T	0.05	0.25	0.16	0.35	STRM			Cdx,Gfi1
15	49446345	0.97	0.99	rs17478694	C	G	0.05	0.25	0.16	0.37	FAT, STRM, SKIN			5 altered motifs
15	49448107	0.97	0.99	rs17478785	A	C	0.05	0.25	0.16	0.37	STRM, GI			p300
15	49450872	0.97	0.99	rs4389093	A	G	0.06	0.26	0.16	0.37	4 tissues	OVRY		

Chr., chromosome. Pos., chromosome position. Ref., reference allele. Alt., altered allele. Freq., minor/altered allele frequencies. The candidate functional variant is in blue.
