# Supplementary material for: A functional variant alters binding of activating protein 1 regulating expression of FGF7 gene associated with chronic obstructive pulmonary disease
Source: BMC Med Genet. 2019 Feb 18;20:33. doi: 10.1186/s12881-019-0761-7 (PMC6380023; doi:10.1186/s12881-019-0761-7)
Supplement: Supplementary file 3 — Table S3. The candidate functional variant lies on binding sites for multiple transcription factors. (PDF 71 kb) [file 12881_2019_761_MOESM3_ESM.pdf]

Supplementary Table 3 The candidate functional variant lies on binding sites for multiple transcription factors.

Position Weight Matrix ID	Strand	Match on:<br>Ref: TATATACACTGGGAAACCAAAACATTTGT**A**TGACTCACTTTGTTGCAATATTTGTTTTA<br>Alt: TATATACACTGGGAAACCAAAACATTTGT**G**TGACTCACTTTGTTGCAATATTTGTTTTA
AP-1_disc3	-	DRTGASTCAY
AP-1_known3	+	VTGACTHA
AP-1_known4	+	MTGASTMAB
AP-2_disc1	-	VTGASTCABB
BAF155_disc1	-	GRTGASTCAS
BATF_disc1	+	KNDVTGASTCA
BCL_disc2	+	MTGASTCAKY
Bach1	+	RBSATGACTCAYSNB
Bach2	+	SRTGACKCAYS
Cdx	+	NAYWRHHARAKHHRTAWA
GATA_disc2	-	SHVTGAVTCA
GR_disc2	-	VTGASTCABN
Irf_disc2	-	DHTGASTCAD
KAP1_disc1	-	NDVTGASTCA
Mef2_disc3	+	AWGASTCAG
Myc_disc3	+	VTGASTCABB
PRDM1_disc2	-	RTGASTCAYH
RXRA_disc3	-	DTKASTMAHH
STAT_disc2	+	VTGANTCAYN
TCF4_disc1	+	VTGASTCABN
p300_disc1	+	VTGASTCABN

Ref. reference sequence, Alt., altered sequence.
