# Supplementary material for: A functional variant alters binding of activating protein 1 regulating expression of FGF7 gene associated with chronic obstructive pulmonary disease
Source: BMC Med Genet. 2019 Feb 18;20:33. doi: 10.1186/s12881-019-0761-7 (PMC6380023; doi:10.1186/s12881-019-0761-7)
Supplement: Supplementary file 4 — Table S4. List of primer sequences for quantitative PCR assays. (PDF 52 kb) [file 12881_2019_761_MOESM4_ESM.pdf]

Supplementary Table 4 List of primer sequences for quantitative PCR assays.

**Name**	**Sequences**
GAPDH-F	TGCACCACCAACTGCTTAGC
GAPDH-R	GGCATGGACTGTGGTCATGAG
ChIP-F	TAGACAAAATGCTATTATACCCTTAAGAG
ChIP-R	AATATAGGCATACCTCAGAGATATTGCA
FGF7-F	TCTTCTGTCGAACACAGTGGTACCTGAGGA
FGF7-R	TTGATTGCCACAATTCCAACTGCCACTGTCCT
rs12905203	
probe1	TGTATGACTCACTTTG
probe2	CATTTGTGTGACTCACTT
primer-F	AATGCTATTATACCCTTAAGAGACTAC
primer-R	TAGGCATACCTCAGAGATATTGCAGGCT

F., Forward; R., Reverse; ChIP., chromatin immunoprecipitation.
