# Supplementary material for: A functional variant alters binding of activating protein 1 regulating expression of FGF7 gene associated with chronic obstructive pulmonary disease
Source: BMC Med Genet. 2019 Feb 18;20:33. doi: 10.1186/s12881-019-0761-7 (PMC6380023; doi:10.1186/s12881-019-0761-7)
Supplement: Supplementary file 5 — List of references in Additional file 1: Table S1. (PDF 70 kb) [file 12881_2019_761_MOESM5_ESM.pdf]

References

1. Pillai SG, Ge D, Zhu G, Kong X, Shianna KV, Need AC, Feng S, Hersh CP, Bakke P, Gulsvik A *et al*: **A genome-wide association study in chronic obstructive pulmonary disease (COPD): identification of two major susceptibility loci**. *PLoS Genet* 2009, **5**(3):e1000421.
2. Cho MH, Boutaoui N, Klanderman BJ, Sylvia JS, Ziniti JP, Hersh CP, DeMeo DL, Hunninghake GM, Litonjua AA, Sparrow D *et al*: **Variants in FAM13A are associated with chronic obstructive pulmonary disease**. *Nat Genet* 2010, **42**(3):200-202.
3. Kong X, Cho MH, Anderson W, Coxson HO, Muller N, Washko G, Hoffman EA, Bakke P, Gulsvik A, Lomas DA *et al*: **Genome-wide association study identifies BICD1 as a susceptibility gene for emphysema**. *Am J Respir Crit Care Med* 2011, **183**(1):43-49.
4. Wan ES, Cho MH, Boutaoui N, Klanderman BJ, Sylvia JS, Ziniti JP, Won S, Lange C, Pillai SG, Anderson WH *et al*: **Genome-wide association analysis of body mass in chronic obstructive pulmonary disease**. *Am J Respir Cell Mol Biol* 2011, **45**(2):304-310.
5. Brehm JM, Hagiwara K, Tesfaigzi Y, Bruse S, Mariani TJ, Bhattacharya S, Boutaoui N, Ziniti JP, Soto-Quiros ME, Avila L *et al*: **Identification of FGF7 as a novel susceptibility locus for chronic obstructive pulmonary disease**. *Thorax* 2011, **66**(12):1085-1090.
6. Cho MH, McDonald ML, Zhou X, Mattheisen M, Castaldi PJ, Hersh CP, Demeo DL, Sylvia JS, Ziniti J, Laird NM *et al*: **Risk loci for chronic obstructive pulmonary disease: a genome-wide association study and meta-analysis**. *Lancet Respir Med* 2014, **2**(3):214-225.
7. Hansel NN, Pare PD, Rafaels N, Sin DD, Sandford A, Daley D, Vergara C, Huang L, Elliott WM, Pascoe CD *et al*: **Genome-Wide Association Study Identification of Novel Loci Associated with Airway Responsiveness in Chronic Obstructive Pulmonary Disease**. *Am J Respir Cell Mol Biol* 2015, **53**(2):226-234.
